# Paloma Giangrande appointed for a second term as the editor-in-chief of *MolecularTherapy Nucleic Acids*

**DOI:** 10.1016/j.omtn.2026.103128

**Published:** 2026-11-01

**Authors:** Betty Schiefelbein

**Affiliations:** 1 American Society of Gene & Cell Therapy, Waukesha, WI, USA

**Figure:**
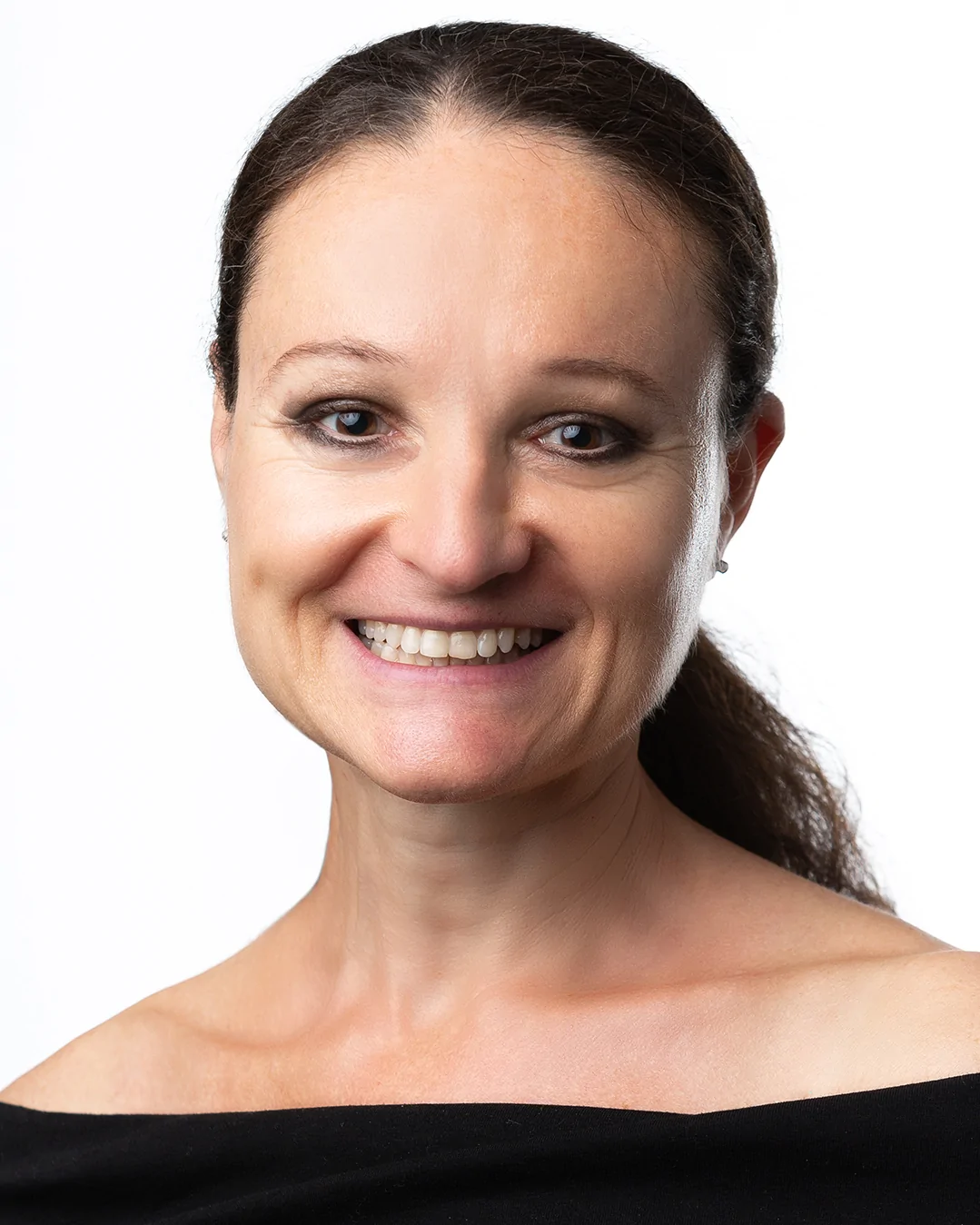
Paloma Giangrande, Editor-in-Chief, *Molecular Therapy Nucleic Acids*

Dr. Giangrande, an internationally recognized RNA pioneer and executive with over 20 years of expertise across academia and industry, has been appointed for a second term as editor-in-chief of *Molecular Therapy Nucleic Acids* (MTNA). She has served as editor-in-chief since 2022, and her second five-year term will begin in January 2027. During her first term, she guided MTNA through an era of unprecedented growth in nucleic acid therapeutics. Under her leadership, the journal expanded its scope to include computational biology and AI/machine-learning applications. She also partnered with the International Society of Nucleic Acid Immunity (NAIS) to integrate the field of innate immune sensing of nucleic acids into MTNA’s scope; as a result of this partnership, MTNA became the official journal of NAIS.

Dr. Giangrande is currently president, co-founder, and chief scientific officer at Program Therapeutics and President of BioLogic Insights, LLC. She previously served as senior vice president of Discovery and Translational Biology at Orbital Therapeutics through its $1.5B acquisition by Bristol Myers Squibb and held key executive leadership roles at Wave Life Sciences, Moderna, and Eleven Therapeutics. As a former associate professor at the University of Iowa and long-standing member of American Society of Gene & Cell Therapy (ASGCT), Dr. Giangrande has published more than 80 peer-reviewed papers with over 11,000 citations and received ASGCT’s Outstanding New Investigator award in 2013.

“I am honored to continue serving the ASGCT community and leading MTNA during such a transformative period for genetic medicine,” said Dr. Giangrande. “Over the next five years, our goal is to proactively scout frontier modalities, including circular RNA, tRNAs, targeted extrahepatic delivery, precision RNA editing, and AI-driven therapeutics, while fast-tracking high-impact discoveries and providing exceptional value to our authors and readers.”

ASGCT and the *Molecular Therapy* editorial team congratulate Dr. Giangrande on her reappointment and look forward to the journal’s continued growth under her leadership.

